# Effects of Segment Length and Crosslinking via POSS on the Calorimetric and Dynamic Glass Transition of Polyurethanes with Aliphatic Hard Segments

**DOI:** 10.3390/ijms242216540

**Published:** 2023-11-20

**Authors:** Konstantinos N. Raftopoulos, Panagiotis A. Klonos, Patrycja Tworzydło, Jan Ozimek, Edyta Hebda, Apostolos Kyritsis, Krzysztof Pielichowski

**Affiliations:** 1 Department of Chemistry and Technology of Polymers, Cracow University of Technology, ul. Warszawska 24, 31-155 Kraków, Polandjan.ozimek@pk.edu.pl (J.O.); edyta.hebda@pk.edu.pl (E.H.); kpielich@pk.edu.pl (K.P.); 2 Department of Physics, Zografou Campus, National Technical University of Athens, Iroon Politechneiou 9, 157 80 Athens, Greece; pklonos@central.ntua.gr (P.A.K.); akyrits@central.ntua.gr (A.K.)

**Keywords:** polyurethane, glass transition, POSS

## Abstract

The glass transition in polyurethanes is a complicated phenomenon governed by a multitude of factors, including the microphase separation, which in turn depends strongly on the molar mass of the hard and soft segments, as well as the presence of additives. In this work, we study the effects of the segments’ length on the microphase separation and consequently on the calorimetric and dynamic glass transition of a polyurethane with aliphatic, “flexible” hard segments. It is found that the dependence of the calorimetric glass transition follows the same principles as those in systems with aromatic hard segments. Strikingly, however, the dynamic glass transition, as studied by dielectric spectroscopy, shows a slowing down of its dynamics despite a decrease in $T_{g}$. This discrepancy is discussed in terms of the strong dielectric response of the flexible segments, especially those close to the interface between the hard domains and soft phase, as opposed to a weak thermal one. In addition, polyhedral oligomeric silsesquioxanes (POSS) are introduced in the soft phase of the three matrices as crosslinking centres. This modification has no visible effect on the calorimetric glass transition; nevertheless, it affects the microphase separation and the dielectric response in a non-monotonic manner.

## 1. Introduction

The glass transition of polyurethanes is quite a complicated issue, for it is governed not only by their chemical structure but also by their morphology, which in turn is governed by a multitude of parameters [1,2,3,4].

Keeping things simple for a moment, the chemical structure of conventional polyurethanes can be visualized as an alternating series of moieties originating from a diisocyanate and a diol, which are interconnected through a urethane group formed upon the polyaddition reaction of the said groups [5]. More often than not, the design of polyurethanes involves two kinds of diols: a long oligomeric one playing the role of a flexible component; and a short one (i.e., a chain extender), the purpose of which is to stick together the usually short isocyanate moieties forming segments rich in urethane groups. The segments originating from the long diol (macrodiol) are referred to as soft segments, and the urethane-rich ones as hard segments. Hard segments tend to form secondary hydrogen bond networks [6,7,8], resulting in a micromorphology of hard microdomains with typical sizes in the order of several tens of nanometres dispersed in a continuous soft phase consisting of soft segments with some diluted hard ones [5,9].

The extent of this microphase separation depends on a multitude of parameters, including the chemical nature and compatibility of the two types of segments, their mass fraction, and the molar mass (“length”) of the hard segments. Moreover, the thermal history of the polyurethanes has a very significant effect. These aspects are quite common knowledge, studied in the last decades of the 20th century in the seminal works by Koberstein’s group and more recently in works by other groups, including ours [2,4,10,11,12,13,14,15,16,17]. As a rule of thumb, shorter hard segments tend to dissolve rather than form hard microdomains, thus reducing the degree of microphase separation. The fast cooling from the melt through the disorder-to-order transition also tends to cause a weaker phase separation, whereas, in the case of the isothermal development of hard microdomains, the annealing temperature plays a very complicated role, leading not only to different degrees of microphase separation but also to different internal ordering within the hard microdomains. The effect is very strongly related to the viscosity of the system [17].

The degree of microphase separation plays a crucial role in the molecular mobility of polyurethanes. Two glass transition events should be expected, one related to the soft phase and one to the hard microdomains. The soft phase can be treated as a blend of soft segments and diluted hard segments. Diluted hard segments slow down the mobility of the soft one and increase the glass transition temperature. The glass transition of hard microdomains is a more complicated issue. The mobility of hard segments is much slower as compared to the macrodiols, due to their rigid structure and the constraints imposed by crosslinking. Thus, they exhibit higher glass transition temperatures, usually above room temperature [18]. However, this glass transition is not very often observed in practice, or it is observed only indirectly through the enthalpy relaxation endotherm associated with it [19,20].

Looking at the issue in more detail, more aspects should be considered. We already pointed out that the typical size of the hard microdomains is in the order of tens of nanometres. Hence, the interface between hard domains and the soft phase should play a significant role in the properties of the materials. Indeed, in earlier research, we and other researchers have detected via dielectric spectroscopy a relaxation α’ accompanying the dynamic glass transition (α relaxation) of polyurethanes and polyureas [21,22,23,24,25,26]. This relaxation has been shown to (i) follow the trends of α relaxation, (ii) merge with it at low frequencies, (iii) have a cooperative (Vogel–Fulcher–Tammann–Hesse) behaviour, and (iv) have a weak, if any, thermal response but a detectable dielectric one. These characteristics are similar to those of a relaxation related to the rigid amorphous fraction formed at the interfaces between nanoparticles or crystallites and bulk polymers [27]. 

In more recent research, however, we studied systems based on linear aliphatic isocyanates, which are essentially more flexible than typical cycloaliphatic or aromatic ones. In the first of these works [28], no standalone relaxation was observed on the low-frequency side of α. In the second one [21], albeit essentially the same system, we detected a very weak one. In both of these cases, the trends of the calorimetric glass transition temperature were not observed in the time scale of α relaxation. This often times can be attributed to the changing cooperativity of the relaxations [29]; however, this was not the case in that work. Instead, there were significant effects on the asymmetry of the single peak associated with the α relaxation. This paradox was discussed in terms of the hypothesis that in such systems, it is not necessary for a distinct interface to be formed. Instead, the transition of the dynamics from the slow mode (near hard domains) to the bulk mode is a smooth one. At the same time, the slower modes do not contribute to the thermal response but only to the dielectric one.

In the aforementioned studies [21,28], as well as in other research [23,24,29,30,31,32], we have studied extensively the glass transition in systems with POSS introduced in polyurethanes with different chain architectures. POSS (polyhedral oligomeric silsesquioxanes) are large molecules with a siliceous, typically cubic, core with Si atoms in the vertices interconnected with oxygen atoms [33,34,35,36,37,38,39]. On each Si atom, an organic group is attached. These organic groups may or may not be reactive. By appropriately choosing the reactive groups, it is possible to introduce POSS moieties in the macromolecular structure itself, by the so-called nanobuilding block approach. Moieties with one reactive group can be incorporated as pendent particles, those with two reactive groups allow for the incorporation of the POSS moiety along the main chain of the polymer, and those with more reactive groups act as branching points/chemical crosslinks [35]. The architecture plays a crucial role in the influence of POSS on the glass transition and on the mechanisms of this influence. We have summarized the effects of the architecture in systems based on an aromatic methylene diphenyl diisocyanate (MDI) in Ref. [1].

Motivated by the previous interesting findings, we continue this line of research in a more systematic effort to understand how the flexibility of the hard segments affects the overall molecular and charge mobility of polyurethanes. For this reason, we synthesized a system based on the “flexible”, linear, and aliphatic hexamethylene diisocyanate (HDI), and compare its properties with our earlier findings on an otherwise identical system based on an aromatic methylene diphenyl diisocyanate (MDI) [16].

The macrodiol in these systems is poly(tetramethylene ether glycol) (PTMG), and the chain extender is 1,4 butanediol (BD); thus, the hard segments of the current system consist of HDI-BD sequences. The ratio of hard to soft segments is 50:50 wt%. In order to study the effects of segment length, we use PTMG with three different molar masses (1000, 1400, and 2000), essentially leading to the average molar masses of the hard segments being the same values. We will show that the general trend of increasing microphase separation/decreasing $T_{g}$ with the increasing molar mass of segments is retained; however, there are discrepancies regarding the time scale of the dynamic glass transition and different distributions of relaxation times, especially with respect to the decelerated dynamics at the interfaces. The results are also discussed in terms of morphology with information drawn indirectly from differential scanning calorimetry and from the Maxwell–Wagner–Sillars-type interfacial effects. 

**Scheme 1:**
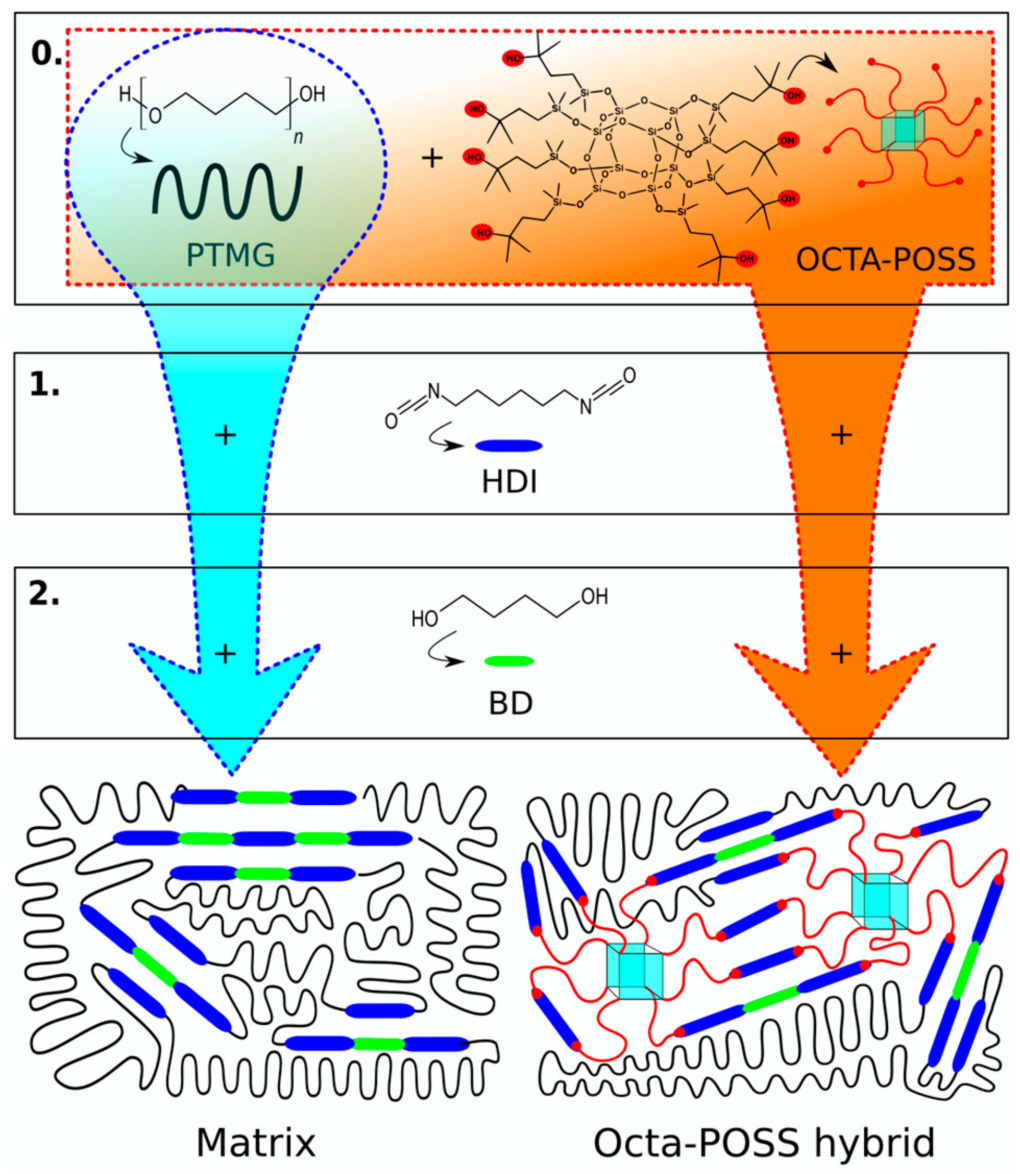
Synthesis route and final chemical structure of the composites. Blue route indicates the synthesis of the matrix, and orange one that of the hybrids. Numbers indicate the steps of the reaction.

At the same time, we are interested in how POSS acting as “heavy” chemical crosslinks affect the glass transition in these multi-component systems. Thus, a POSS moiety with eight hydroxy functional vertex groups is introduced in the system with mass fractions up to 6 wt% (Scheme 1). We have studied a similar system based on an aromatic diisocyanate in the past [24], but with a crucial difference: in the current system, POSS replaces macrodiol, whereas in the previous system, it replaced hard segments. In other words, here the system with 6 wt% POSS consists of 50 wt% hard segments (HDI-BD sequences), 44 wt% macrodiol, and 6 wt% POSS, whereas the previous system with the same POSS concentration consists of 44 wt% hard segments (MDI-BD sequences), 50 wt% macrodiol, and 6 wt% POSS. Here, we study the effects of POSS on the glass transition of these three matrices and compare the results to those using the “aromatic” system, taking into account this difference in composition.

## 2. Results

### 2.1. Glass Transition of the Matrices

#### 2.1.1. Differential Scanning Calorimetry (DSC)

**Figure 1:**
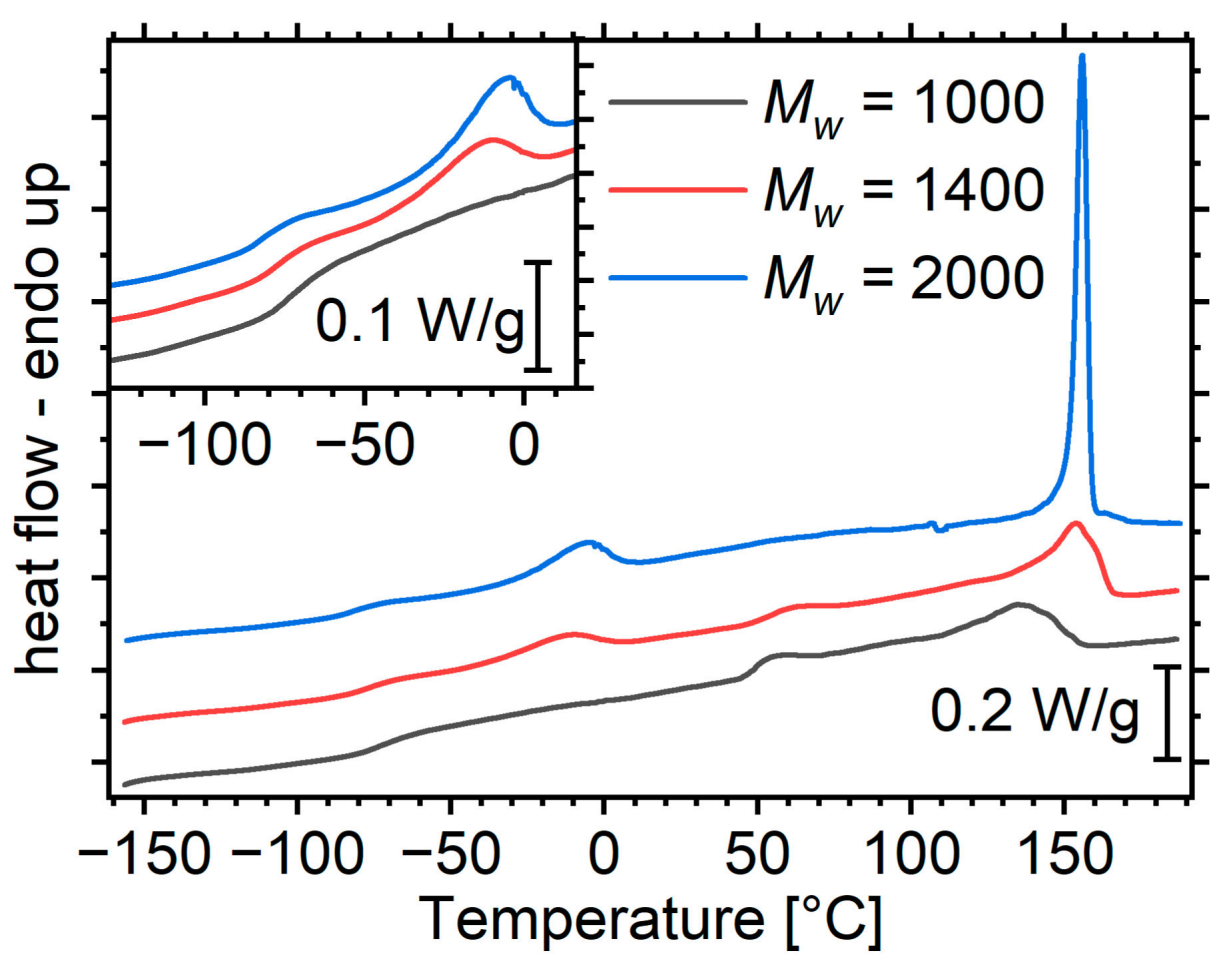
DSC heating curves of the matrices, normalized to sample mass. The inset shows a magnification in the regions containing soft-phase-related phenomena. The curves have been translated for clarity.

The DSC curves of the materials in this study can be divided in two distinct regions (Figure 1). The sub-ambient region contains phenomena originating in the soft phase, whereas at higher temperatures, hard-domain-related phenomena occur. We will describe and comment on them in order of increasing temperature.

##### Soft-Phase Glass Transition

**Figure 2:**
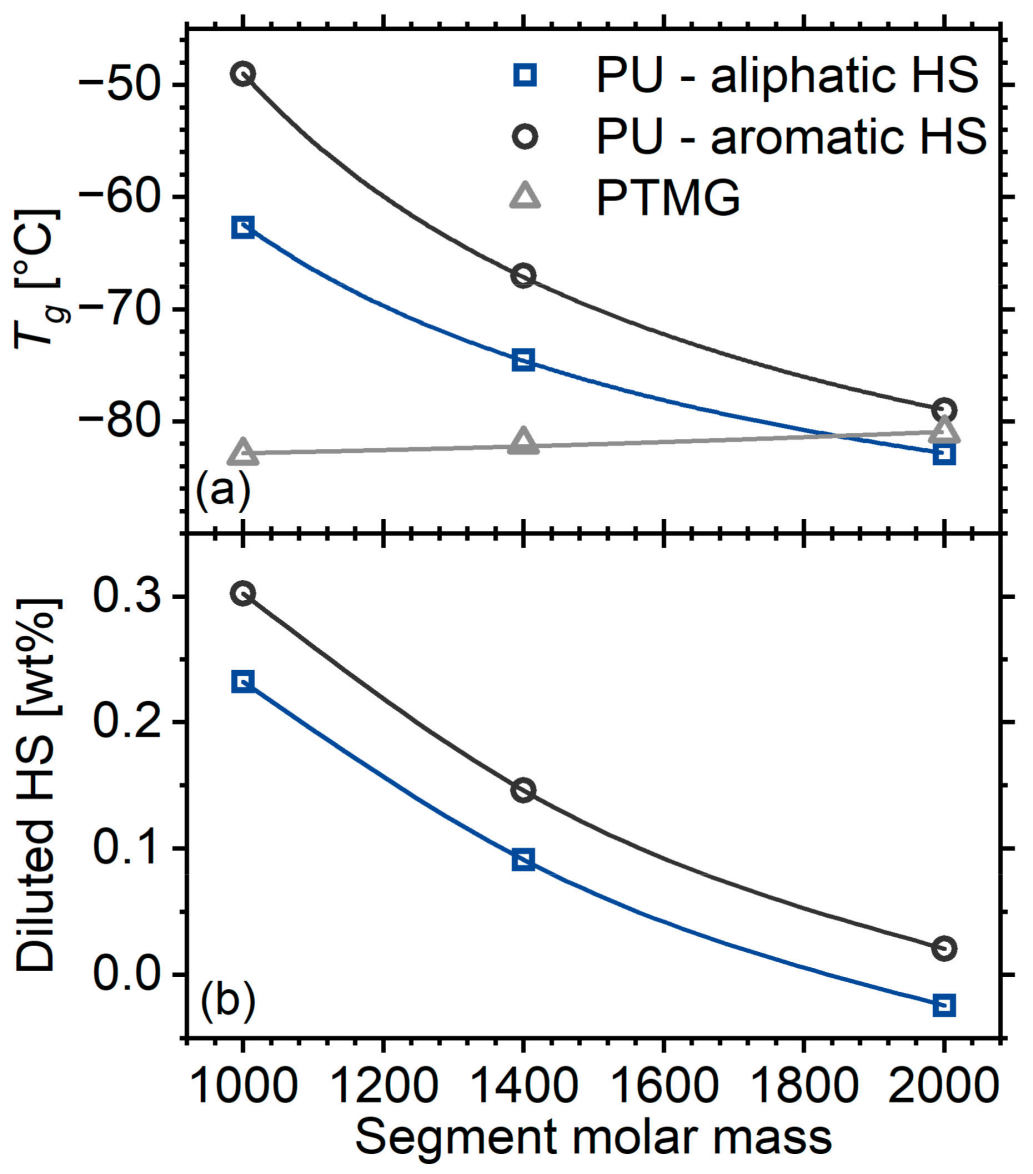
(**a**) Calorimetric glass transition temperature of the polyurethane matrices as a function of segment molar mass. Data are also included for a system identical to the one at hand but with MDI-based (rigid) hard segments and macrodiols taken from reference [16]. (**b**) Concentration of diluted hard segments in the soft phase as calculated by Fox’s model.

The soft-phase glass transition manifests itself as an endothermic step at the temperature region below −50 °C (Figure 1, inset). The glass transition temperature, $T_{g}$, of the matrix with the shortest segments is approximately 20 K above that of the corresponding macrodiol (Figure 2a). With increasing molar mass of the segments and the corresponding increase in the degree of microphase separation, the step migrates to lower temperatures, reaching the value of the macrodiol. This is a result that is well documented in the literature and is also described in the introduction [2,16]. The $T_{g}$ of the systems at hand are several K lower than that of the polyurethanes prepared in an identical manner, but with an aromatic, rigid diisocyanate (MDI) [16]. This originates from two mechanisms. The first is that the diluted hard segments in the current work are more flexible. Indeed, it has been reported that the $T_{g}$ of the HDI-BD homopolymer lies around 50 °C [18], whereas that of MDI-BD lies around 108 °C [19]. The second possible mechanism is that the HDI-based PUs exhibit a better microphase separation, as expected based on the literature [40]. Assuming the validity of Fox’s law [41] and based on the $T_{g}$ values of the macrodiol from Ref [16], it is possible to provide a rough estimation of the amount of hard segments diluted in the soft phase (Figure 2b). The results indicate that aromatic hard segments tend to dissolve better than their aliphatic counterparts in the soft phase.

##### Melting of Soft Segments

This manifests as a broad endothermic peak around −10 °C and occurs for the materials with segment molar masses of 1400 and 2000; it is more intense for the latter (Figure 1). It is expected in general that longer chains can order more effectively than short ones. In addition, diluted hard segments interrupt the ordering of the soft ones; therefore, the longer both segments are and the more phase separation, the more effective the ordering is [2,16]. In the system with MDI-based hard segments, crystallization was observed only at the highest segment molar mass, indicating either a weaker disruption of the ordering by the aromatic rings as compared to the linear aliphatic chain, or a lower degree of microphase separation.

##### Relaxation of Hard Microdomains

PUs with segment molar masses of 1000 and 1400 exhibit an endothermic event around 50 °C. Most polyurethanes exhibit a broad peak in this region which is generally attributed to relaxations of the hard microdomains [4,17]. This peak is often referred to as the annealing peak because it typically occurs 20–40 K above the temperature at which such materials undergo annealing [17]. It has been suggested that it corresponds to the enthalpy relaxation accompanying the glass transition of the hard microdomains [20]. The lack of a corresponding step is often explained by a negligible $\Delta c_{P}$ value. In the case of the HDI-BD homopolymer though, a glass transition event has been reported in this region [18], and the curves of Figure 1 indeed clearly show a step, at least for the lowest segment molar mass, corroborating the attribution of the event to a glass transition event.

The event is more pronounced for the matrix with the shortest segments. This should be related to the fact that its hard domains are less crystalline than its longer-segment counterparts (details are provided in the next paragraph). As the glass transition is a phenomenon of amorphous matter, it is essentially more intense in materials with a greater amorphous fraction.

##### Order–Disorder Transitions/Hard Domain Melting

At temperatures above 100 °C, strong endothermic peaks correspond to the gradual disordering of hard microdomains. These peaks in general are complex, consisting of at least two components of controversial origins. We refer the reader to the works by Koberstein’s group [10,12,13] and others [42,43], as well as to an earlier work of ours, in which we summarize the relevant literature [17]. Here, we would like to point out that several phenomena contribute to these peaks: depending on their chemical nature and molar mass, hard microdomains may be crystalline or amorphous. Except for the microphase mixing peaks, crystalline hard domains also exhibit a melting peak. Such an event is expected here: HDI-POSS hard segments are known to exhibit some crystallinity at a molar mass as low as 1000 [28]. Longer segments are expected to exhibit an even higher tendency to crystallization. 

Indeed, with increasing segment molar mass, the high-temperature peaks become progressively narrower, and migrate to higher temperatures (Figure 1), indicating a more intense ordering at the nano- and the atomic scale. It is noteworthy that for the highest molar mass, the peak is unusually sharp for this type of transition, resembling a melting event rather than a microphase mixing one. This observation, combined with the almost complete lack of a glass transition step for PU2000, as described in the previous paragraph, points to the rather safe assumption that hard domains of PU2000 are almost completely crystalline.

#### 2.1.2. Molecular Mobility on the Matrices

**Figure 3:**
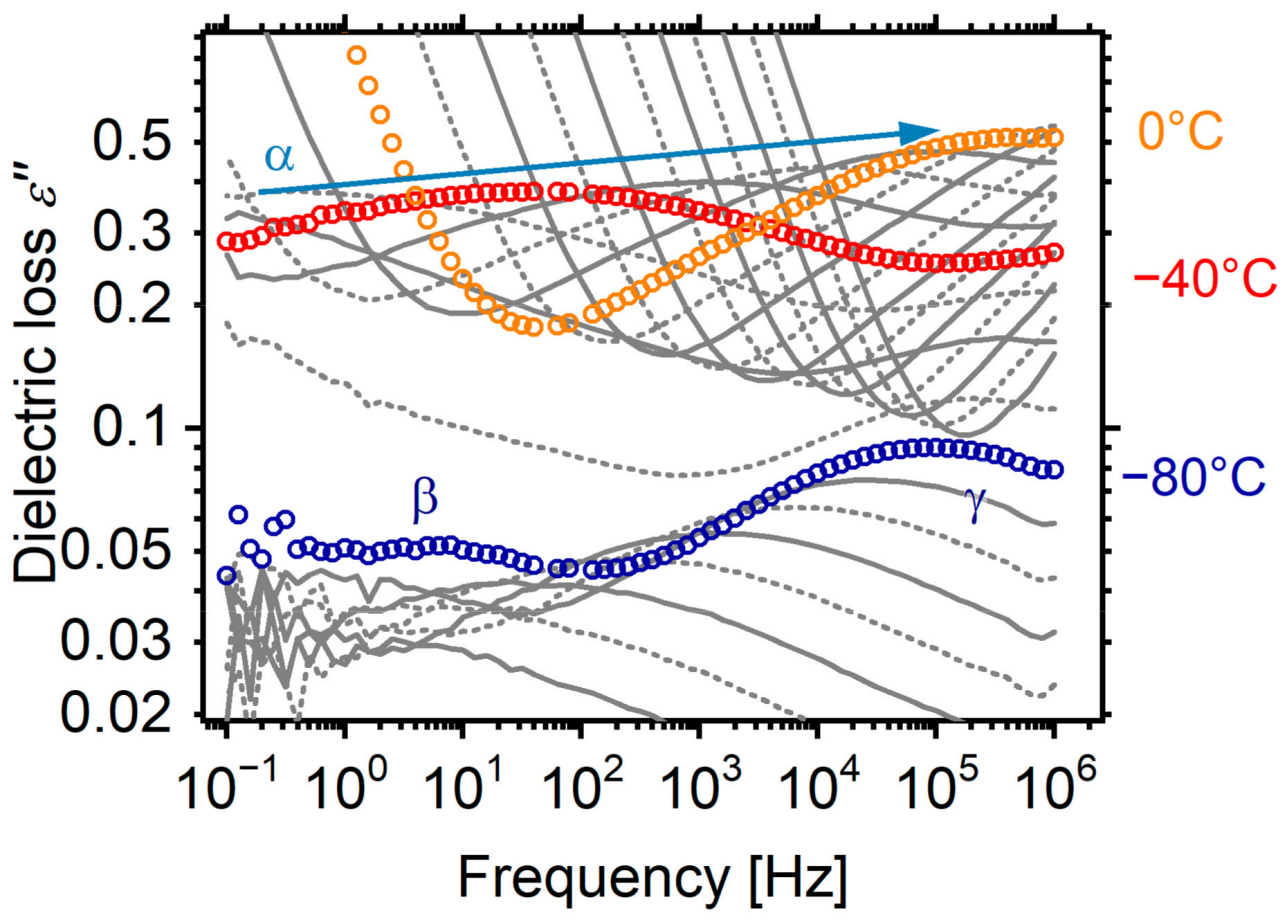
Dielectric loss spectra of PU1000, as a representative sample. The spectra are shown at steps of 10 K and key temperatures are annotated on the plot.

Before we proceed to a formal analysis of the dielectric spectra, we would like to draw as many conclusions as possible directly from the raw data. The dielectric spectra of the material with the short segments (PU1000) have been reported in two of our earlier articles [21,28]. However, we repeated the experiments to eliminate any differences that might have occurred by slight changes in the preparation (e.g., slightly different thermal histories, shapes of reactors, etc.) that are known to have a significant effect on the properties of polyurethanes. The raw dielectric results are presented and analysed in the form of the imaginary part of dielectric permittivity *ε*″ against the frequency *f*. *ε*″ is considered to represent the dielectric loss; moreover, the various dipolar motions are recorded as relaxation peaks in the *ε*″(*f*) spectra at various temperatures. The spectra of PU1000 are very typical for polyurethane elastomers (Figure 3), but we would like to describe the landscape before we proceed to the more interesting spectra of the materials with longer segments. At low temperatures, two secondary relaxations are recorded, corresponding to the crankshaft motion of methylene sequences along the polyether contour (γ relaxation) [44] and the motion of the carbonyl of the urethane bond, probed by attached water molecules (β relaxation) [45]. At around −50 °C, the α relaxation associated with the dynamic glass transition enters the experimental window from the low temperature side and migrates to higher frequencies with increasing temperature. At even higher temperatures, the spectra are dominated by a steep slope mainly corresponding to long-range free-charge mobility, i.e., dc conductivity. In the following, we will show that at a closer look, the landscape is somewhat more complicated and is differentiated for the materials with longer segments.

**Figure 4:**
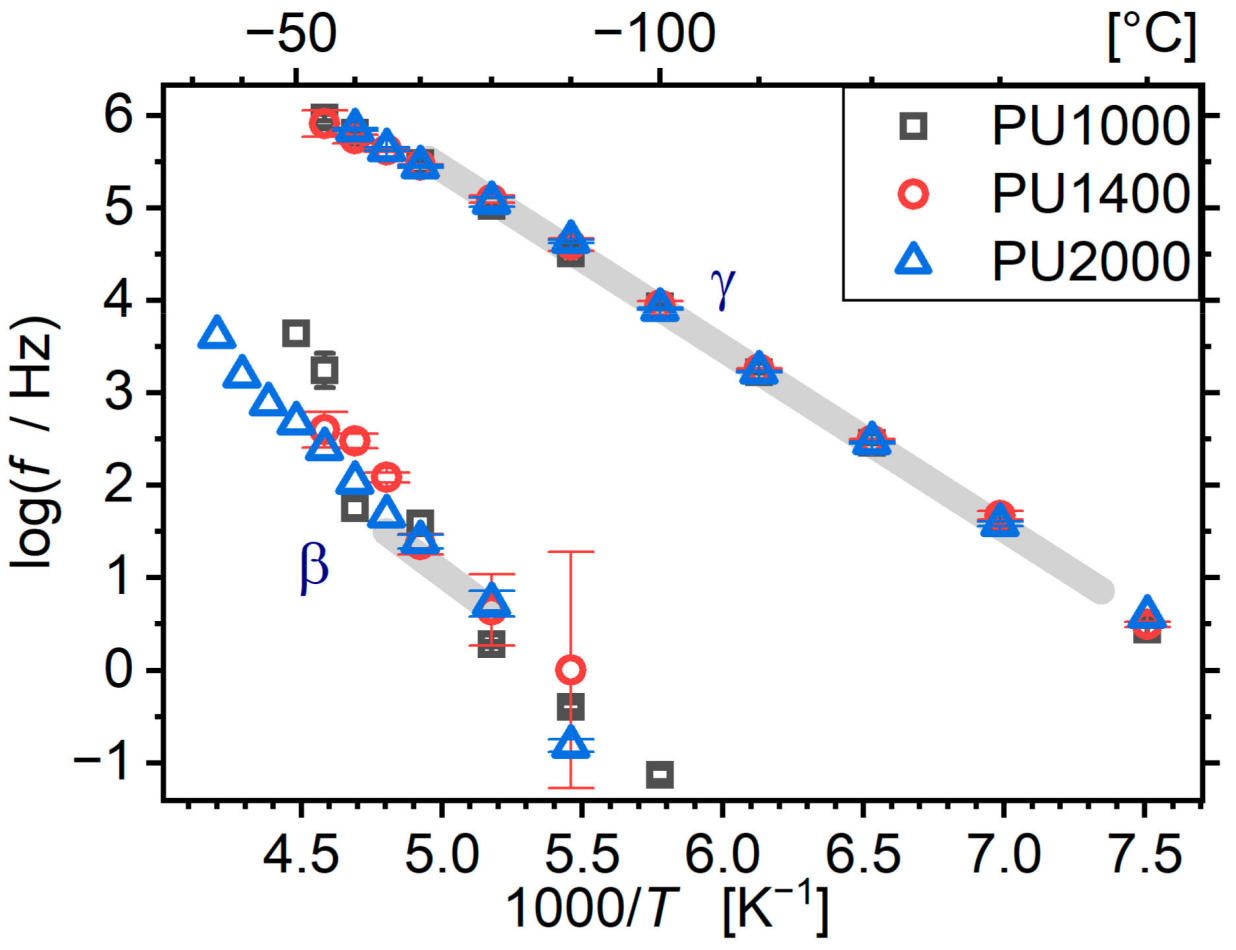
Arrhenius plot for the secondary relaxations of the matrices. Grey bands show the location of the relaxations in the MDI-BD matrix with segmental molar mass of 1400 as reported in [22].

For the sake of completeness, we would like to mention that a trivial fitting procedure (similar to that in Ref [22]) with two Cole–Cole [46,47] terms was performed in the region of the local dynamics. The resulting characteristic frequencies as a function of temperature are plotted in the Arrhenius plot of Figure 4. The molar mass of segments has no effect on the dynamics of the secondary relaxations. In addition, the said dynamics coincides with that of the materials prepared with an aromatic isocyanate. This is due to the local character of the relaxations, which means that the dynamics is affected only by very-small-range interactions in the nearest-neighbour length scale. The γ relaxation, originating on the polyether itself, is not sensitive to any changes in the system. The β relaxation may exhibit some small changes depending on the local density around urethane groups [29,48]. Nevertheless, no such differences are observed here with respect to a system based on an aromatic isocyanate. 

**Figure 5:**
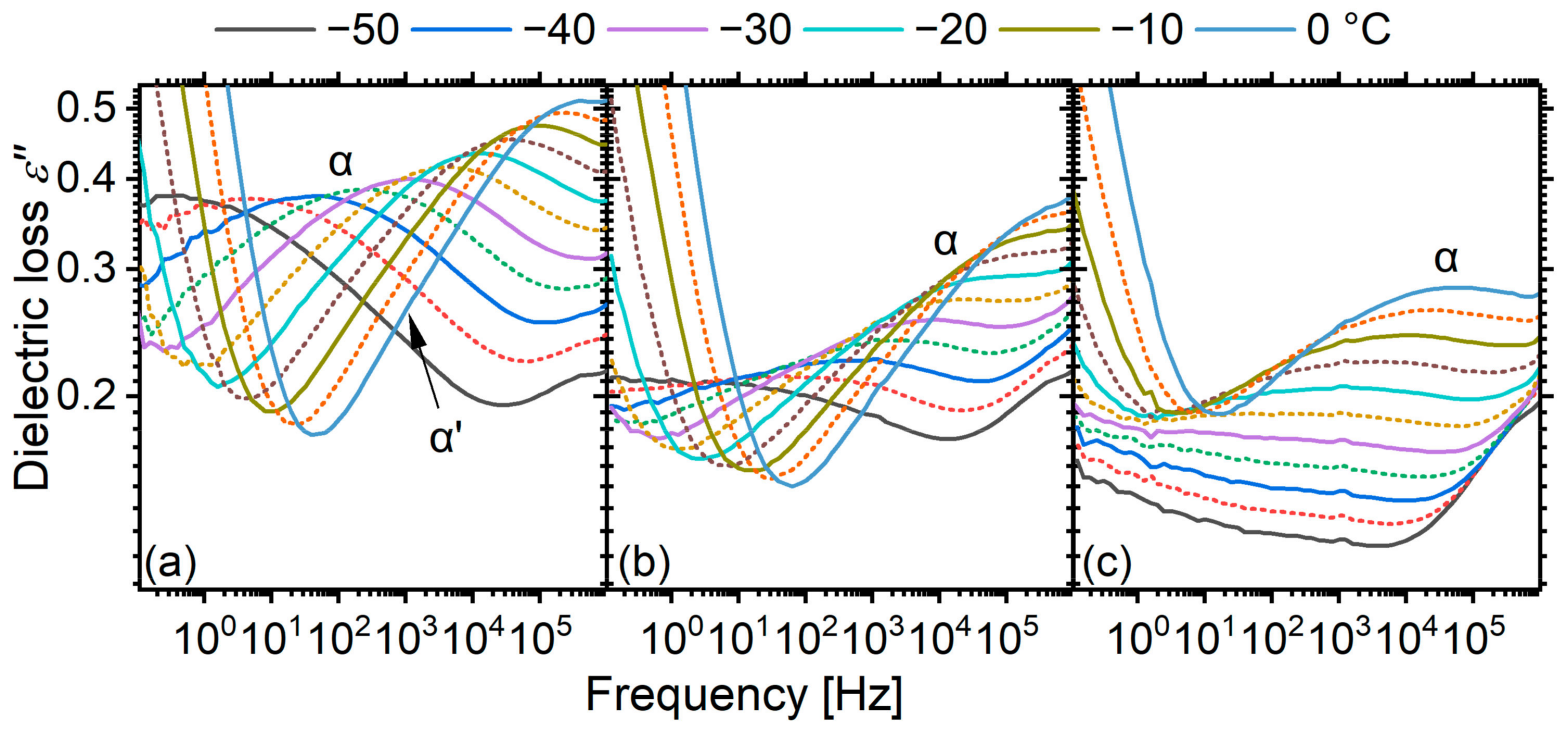
Dielectric loss spectra of the three matrices in the region of the α-relaxation–dynamic glass transition. Spectra are shown in steps of 5 K, but only selected ones are shown in the legend. (**a**) PU1000, (**b**) PU1400, (**c**) PU2000.

Marked changes are observed with changing segmental molar masses on the temperature evolution of α relaxations (Figure 5). The matrix with the longest segments (PU2000) shows a peculiarity (Figure 5c): a well-discerned peak which can be assigned to the α relaxation appears only above −40 °C, at which the melting of the soft phase starts. This is consistent with a transition of the material from the immobile crystalline phase to the mobile amorphous one. However, it is not clear where the α relaxation lies at temperatures below −40 °C. Given that, e.g., at −50 °C, the temperature is already 30 K above $T_{g}$, the relaxation should be visible in the experimental window. However, no well-discerned relaxation peak is visible. Moreover, there is a discrepancy between the dielectric spectra and the thermal glass transition. It is clear that the α relaxation of PU2000, above the melting region, is slower than that of PU1000 and PU1400 (Figure 5), but the calorimetric $T_{g}$ is 30 K lower. A similar gradual increase in the strength of α relaxation is also observed, to a much lesser extent, for PU1400, which also undergoes melting in this temperature range.

Cases in which the peak corresponding to the α relaxation is not well discerned in the dielectric loss spectra are not unheard of in the literature. A notable example is poly(methyl methacrylate) [49,50], whose α relaxation peak is broad enough to span practically the whole experimental window, but it is readily visible as a sharp peak in the so-called isochronal plots, i.e., ε″ as a function of temperature. We have observed the very same case in pure PTMG, which here constitutes the soft segments of the material [16]. We have attributed that to the very high apparent activation energy, which results in a close-to-vertical dispersion band in the Arrhenius plot. Consequently, the relaxation peak is quite broad when the frequency domain is scanned and reasonably narrow when the temperature domain is scanned.

**Figure 6:**
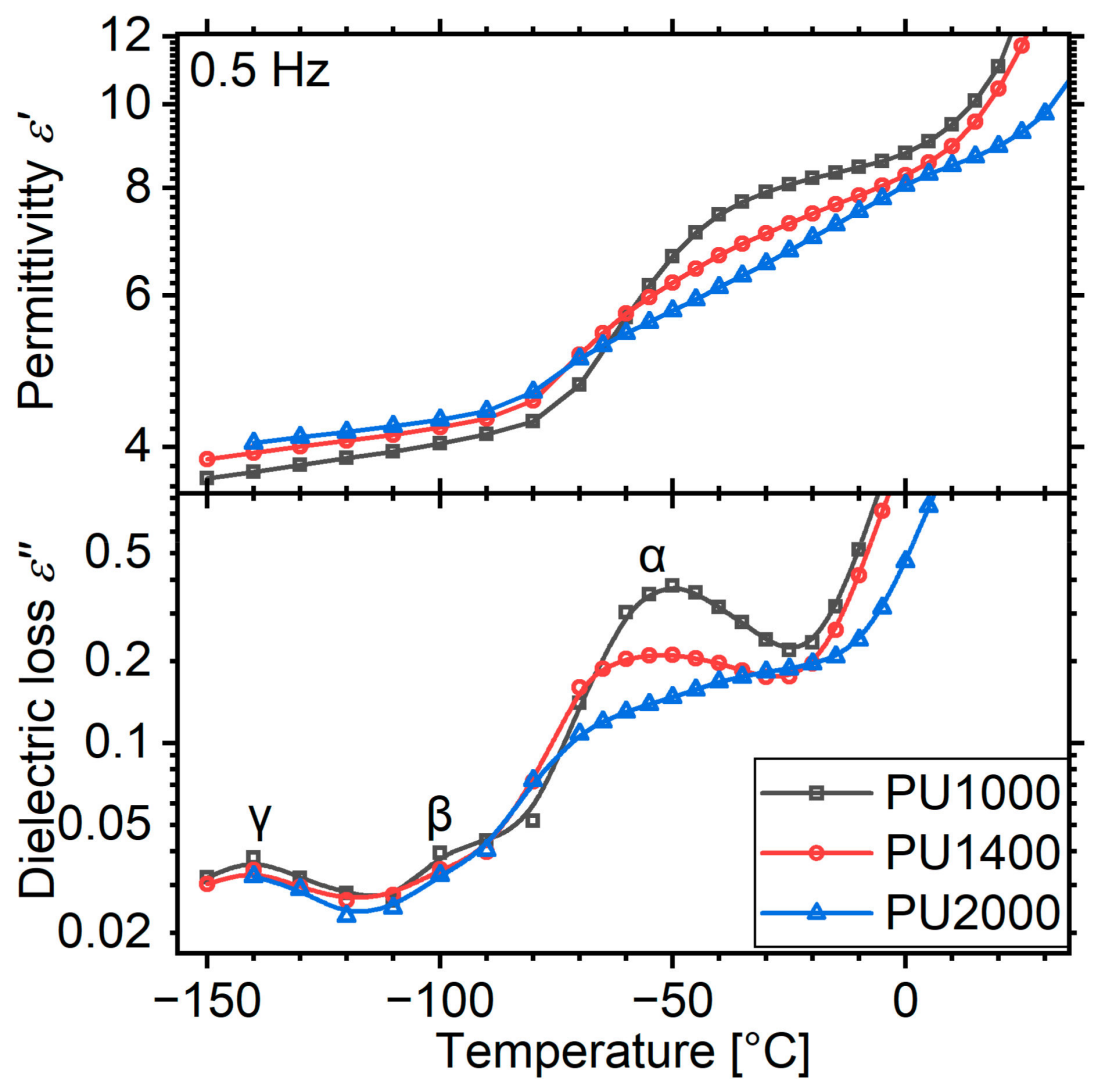
Isochronal dielectric loss plots for the three matrices at a low frequency of 0.5 Hz. The curves have been drawn by replotting data collected isothermally, as a function of temperature.

For this reason, we replotted the data recorded isothermally at a frequency of 500 mHz, close to the low-frequency end of the experimental window (0.5 Hz, Figure 6). Indeed, an increase in ε″ is already observed for PU2000 at temperatures around −80 °C, very close to the calorimetric $T_{g}$. The real part ε′ of the permittivity shows two distinct steps: one on the initiation of the dynamic glass transition and the other upon melting. The first one corresponds to the α relaxation/dynamic glass transition and the second one to the melting, which increases the amount of the amorphous phase that is able to polarize. PU1000, as expected, lacks such a melting feature, and PU1400 shows a very weak one. Up to the point of melting, the $\varepsilon^{\prime }$ curves are in full agreement with the calorimetric glass transition both in terms of temperature and strength. In agreement with the previous observations, the $\varepsilon^{\prime \prime }$ curve of PU2000 does not form a clear peak, but rather keeps increasing as a result of melting.

**Figure 7:**
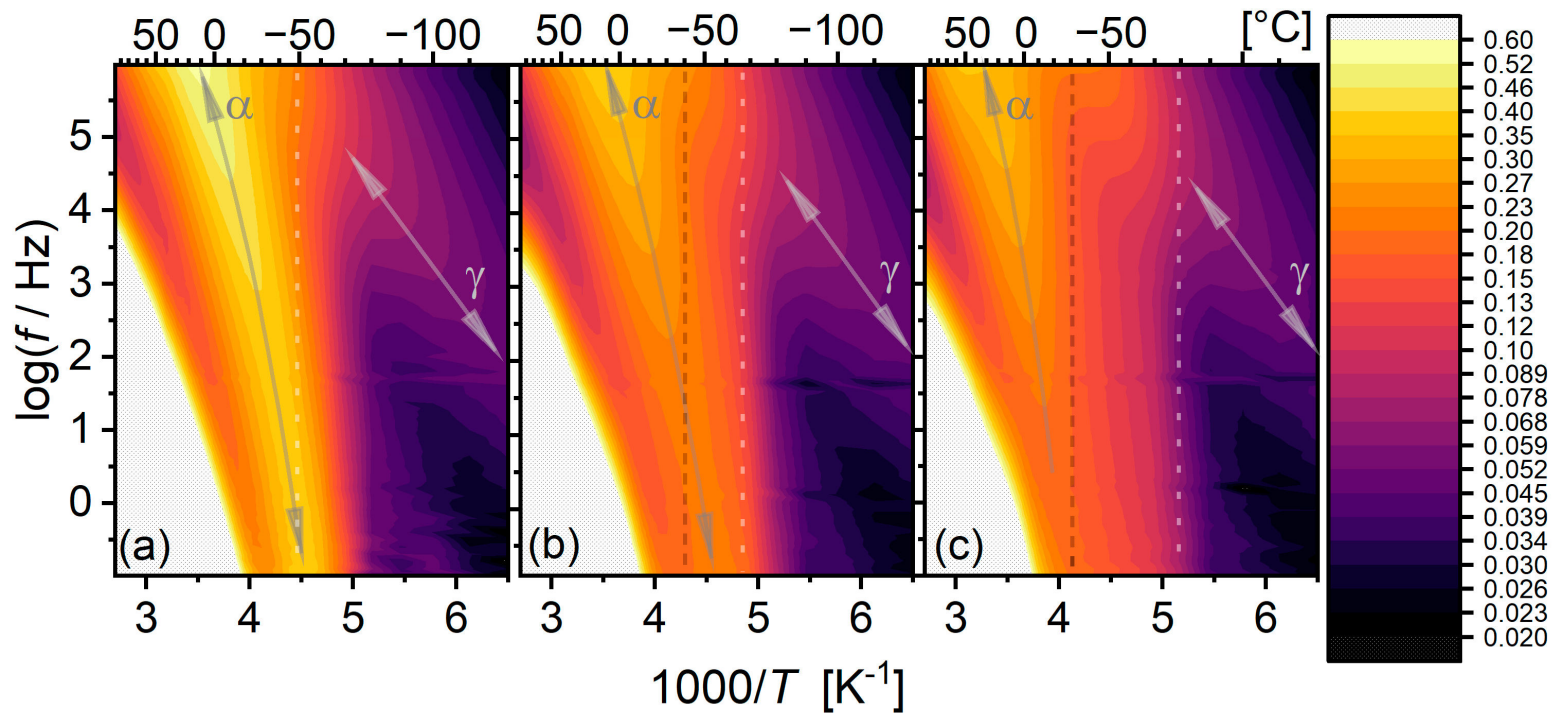
Dielectric loss ε″ heat maps for the matrices under investigation on Arrhenius axes. (**a**) PU1000, (**b**) PU1400, and (**c**) PU1400. Arrows indicate the relaxation bands. Vertical grey dashed lines indicate approximate melting onset as determined via DSC. Vertical, white, dotted lines indicate the position of the calorimetric $T_{g}$. Colour levels are logarithmic and the same for all three maps, according to the colour scale on the right.

In order to better understand the interplay between the time scale and temperature, we drew heat maps of the dielectric loss ε″ on Arrhenius axes (Figure 7). In this representation, relaxations appear as bright bands. For PU1000, the α relaxation band is very well defined and rather narrow, spanning the whole window. Its trace shows the expected concave form, approaching the calorimetric $T_{g}$ on the low-temperature side. On the other end, the α relaxation band of PU2000 is more complicated: the band clearly has already started from the calorimetric $T_{g}$. However, its shape and strength change in a complicated manner throughout the melting event. Eventually, above the melting onset, as observed with DSC, its trace is more clearly observed and is clearly located at higher temperatures/higher frequencies than those of the materials with a lower segmental molar mass. The reason for this is yet to be clarified. The α relaxation band of PU1400, as expected, shows an intermediate behaviour between those of PU1000 and PU2000.

Before proceeding to the results of a model fitting analysis, we would like to draw the attention of the reader to the low-temperature side of the α relaxation (Figure 5a in particular). At higher temperatures, when the peak is on the high-frequency side of the experimental window, it is possible to discern a very weak shoulder. As discussed in the introduction, this is attributed to a relaxation that is routinely observed in polyurethanes and polyureas [22,23,24,25,26] as well as in semicrystalline PTMG [16]. There is some controversy about its origin, but we have provided evidence that it is related to the decelerated cooperative dynamics [21] of polyether chains anchored on structures with negligible internal mobility, much like the dynamics of the so-called rigid amorphous fraction [27,51]. Such structures could be nanoparticles [23], hard domains [24], crystallites [16], etc. This relaxation was mostly observed in systems with aromatic hard segments. In a system similar to those studied here based on flexible aliphatic hard segments, we were not able to detect this relaxation as a standalone peak [28]. However, the α relaxation showed a very particular shape, which could be modelled with a single, yet significantly asymmetric, Havriliak–Negami peak function. We then postulated that while the dynamics is decelerated around the hard domains, due to their higher flexibility, the transition is in general smoother, not forming a standalone relaxation peak. In a subsequent article though, studying a similar matrix [21], we were able to detect a very weak standalone contribution, and by an analysis of the first derivative of ε′, we were able to follow it in a broad temperature range.

In the case at hand, we were able to adequately model the spectra of the matrices in this region with two Cole–Cole model functions [46,47]:

(1) $${\varepsilon (f)}^{*}={\varepsilon (f)}^{\prime }-i{\varepsilon (f)}^{\prime \prime }=\varepsilon_{\infty }+\frac{\Delta \varepsilon }{1+{\left(i\frac{f}{f_{0}}\right)}^{a}}$$

**Figure A1:**
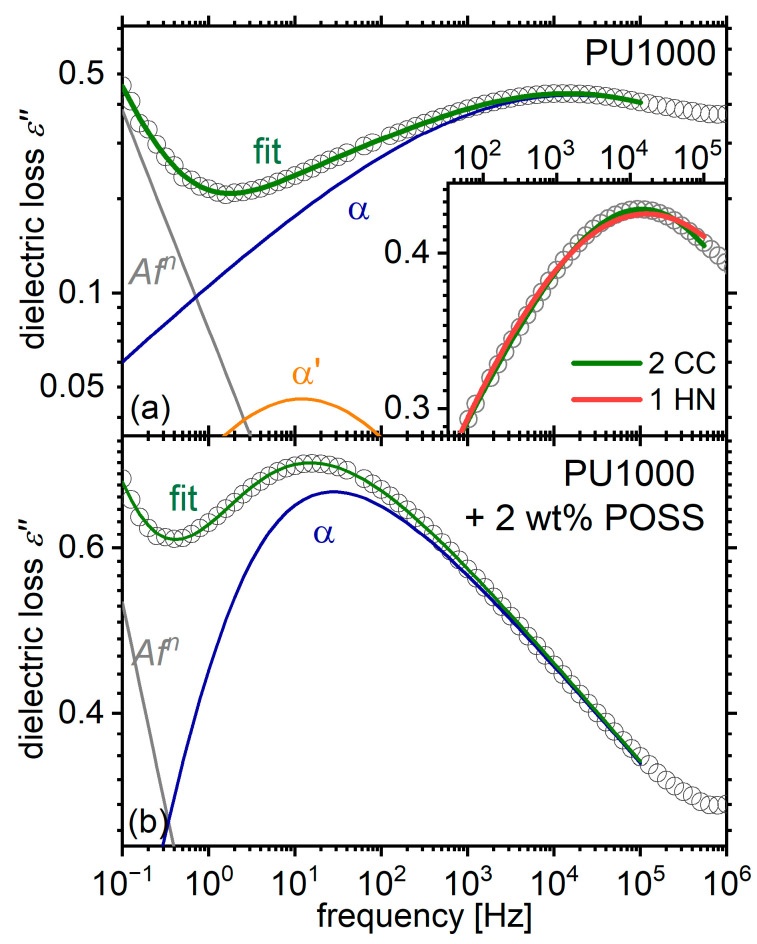
Examples of fitting in the region of α relaxation, for (**a**) the matrix PU1000 and (**b**) its composite with 2 wt% POSS at −20 °C. The inset of panel (**a**) shows a magnification of the tip of the peak, along with fits with two Cole-Cole (CC) model functions (the one used in this article) and with a single asymmetric (Havriliak-Negami, HN) model function. It is evident that the first approach fits the data better. This is not the case for the composite, where a single Havriliak Negami fits the data very adequately.

In which Δε is a strength parameter quantifying the contribution of the relaxing type of dipoles to the static permittivity of the material; and $f_{0}$ is a characteristic frequency that does not necessarily coincide with the peak frequency. Exponent a quantifies the symmetric broadening around $f_{0}$ with respect to the single-relaxation time model (Debye), for which a=1. In the usual case in which there is a distribution of relaxation times, 0<a<1. A representative fitting is shown in Appendix A (Figure A1a).

A power law was used to account for the contributions of charge transport phenomena on the low-frequency side of the spectrum, and the high-frequency range was omitted, in order to exclude the influence of secondary relaxations.

For PU1400 and PU2000, fitting was successful only above their respective melting temperatures, because the large widths of the relaxations and ongoing morphological changes resulted in ambiguous results.

**Figure 8:**
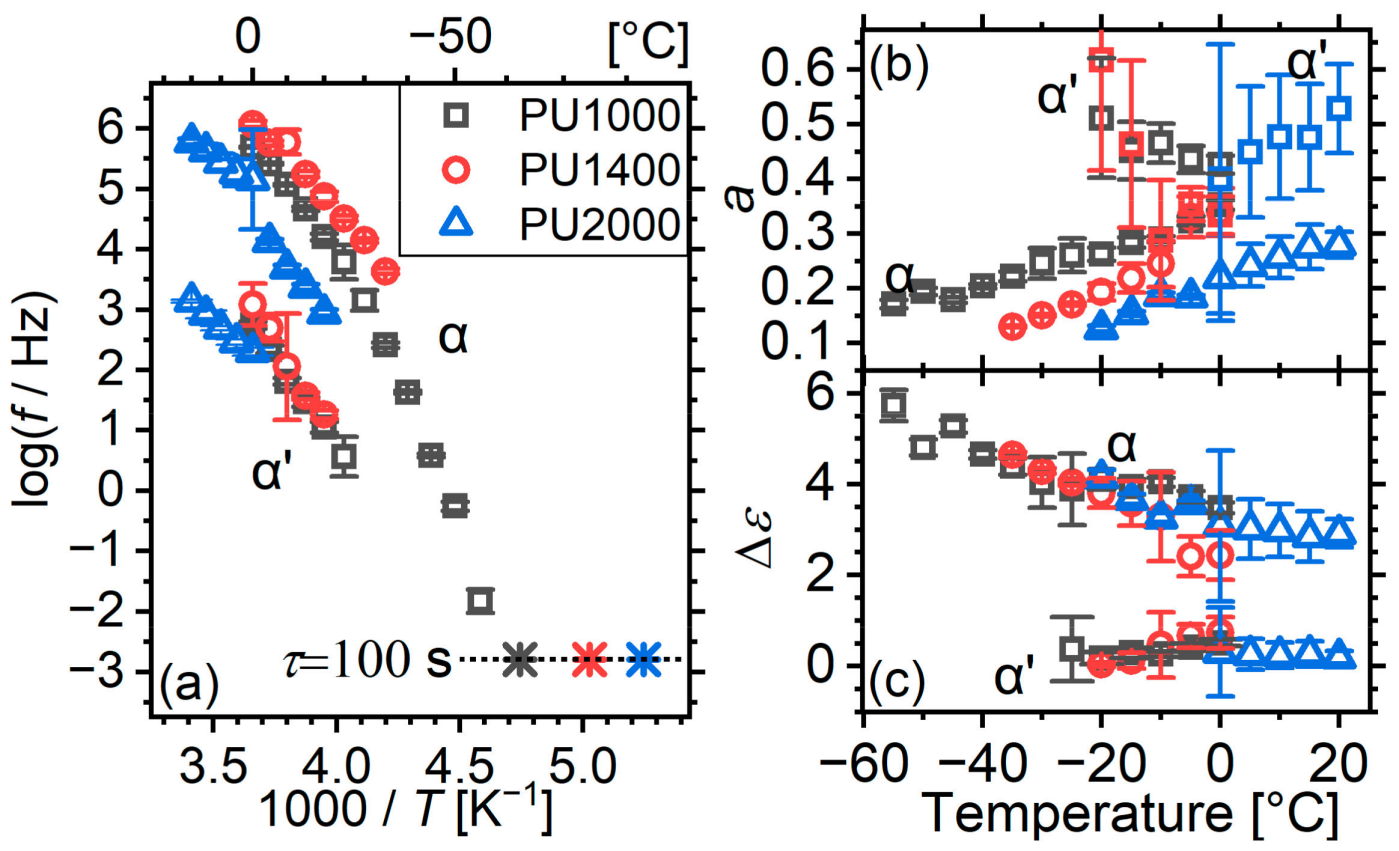
(**a**) Arrhenius plot, (**b**) broadening exponent a, and (**c**) relaxation strength Δε as a function of temperature for α and α’ relaxations of the matrices. Exponent a is not shown for α’ due to its high inaccuracy, which limits our clarity. In (**a**), calorimetric glass transition temperatures are plotted at the equivalent relaxation time of 100 s as stars [54].

The traces of α relaxation of all matrices show the concave form expected for cooperative relaxations (Figure 8a) [52,53]. The relaxation of PU1000 agrees very well with its calorimetric $T_{g}$. Despite the limited range in which the relaxation of PU1400 was detected, dynamic and calorimetric experiments agree in that the relaxation becomes slower with increasing segmental molar mass. This agreement does not hold for PU2000. Dynamic experiments show a slower dynamic glass transition than those of the other matrices, whereas the calorimetric glass transition temperature is lower. We have established so far that dynamic experiments refer to a molten soft phase, whereas the calorimetric one refers to a semicrystalline one. Such radical morphological changes obviously affect the segmental dynamics. However, it would be expected that the raising of constraints with melting would actually accelerate the mobility. Why upon melting the mobility slows down remains undetermined at this point. The answer should probably take into account the quite different state of hard domains in the three matrices, as evidenced via DSC: for low segmental molar masses, hard domains are rather amorphous and glassy, but for high molar masses, they are mostly crystalline. The latter, upon the melting of the soft phase, may play a peculiar role in mobility. For a more concrete understanding of the phenomena, morphological studies (e.g., small-angle X-ray scattering) should be conducted at low temperatures in order to study in detail the changes in the admittedly very complex micromorphology during the melting of the soft phase.

The relaxations are very broad, with broadening exponents a even below 0.2 (Figure 8b). This is an indication of a quite high level of inhomogeneity. For comparison, the system based on the aromatic MDI-BD hard segments exhibited broadening exponents in the range of 0.76–0.79 [16]. Apparently, the more flexible HDI-BD segments participate more in molecular dynamics and increase the inhomogeneity. With increasing segmental molar mass, the broadening increases too (a decreases), which is somewhat counterintuitive given the more complete phase separation. With increasing temperature, the width decreases, which is an indication of gradual homogenization.

The broadening exponent of the α′ relaxation is somewhat higher than that of α; however, due to significant scattering and inaccuracy, it is not possible to extract any further information from it.

The strength Δε of α relaxation is similar for all matrices (Figure 8c). This is also somewhat surprising. Δε depends on the concentration of the polarizable material and its polarizability. Assuming that the degree of microphase separation is higher for higher segmental molar masses, as expected from earlier works, Δε should decrease. This is what we had observed in the system based on MDI-BD hard segments [16]. Interestingly however, in this earlier work, the observed Δε was rather in the order of 1.3 (for a segment molar mass of 1000) to 4.0 (for a segment molar mass of 1000). A hypothesis to understand this result is that the more flexible HDI-BD hard segments have a much higher level of polarizability and dominate the response. This might also be related to the discrepancy with the slower time scale of the α relaxation of PU2000 as compared to its shorter-segment counterparts.

### 2.2. Charge Transport in the Matrices

**Figure 9:**
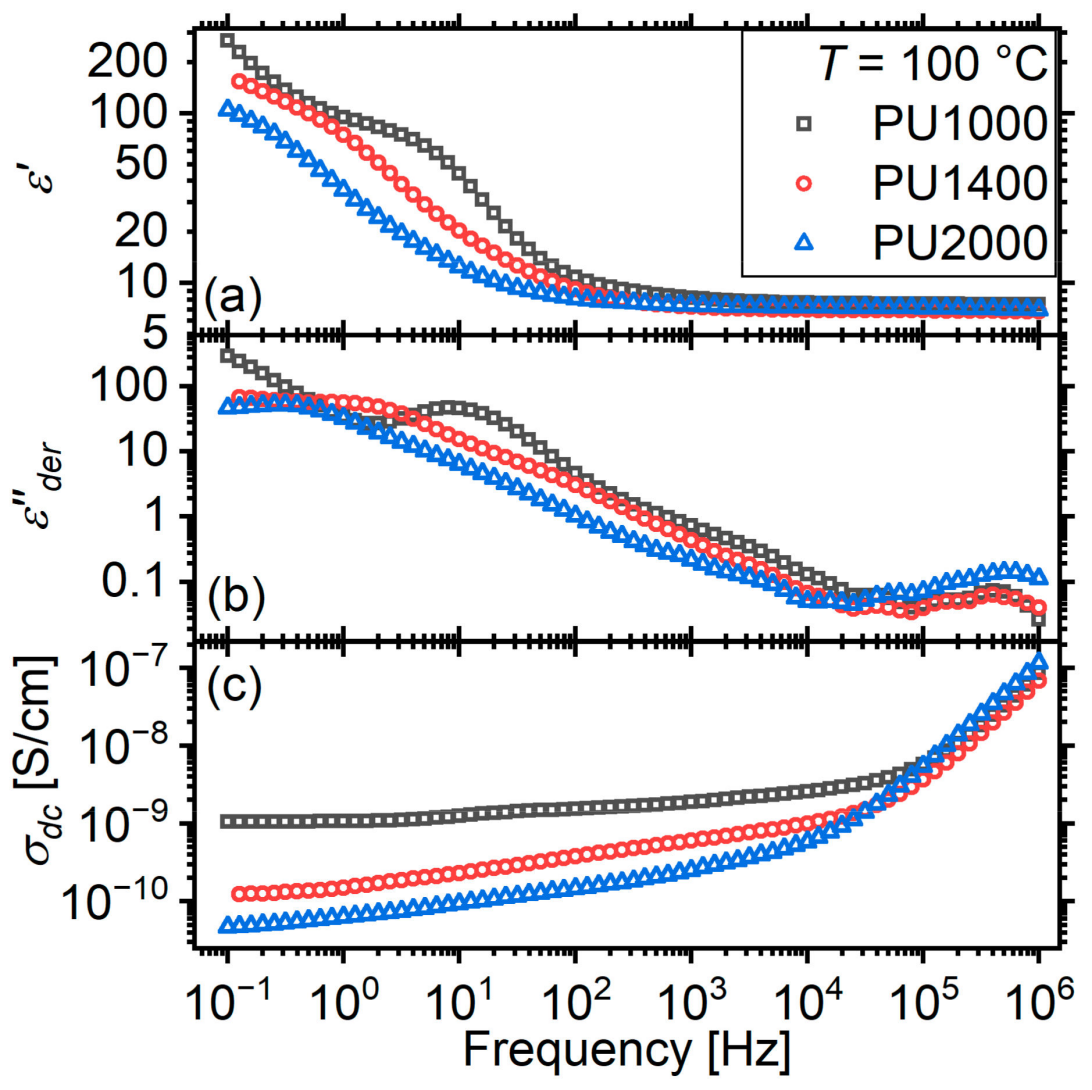
(**a**) real part of dielectric function ε′, (**b**) conductivity-free (details are provided in text) dielectric loss $\varepsilon_{der}^{\prime \prime }$, and (**c**) real part of conductivity σ′ spectra of the three matrices at temperature of 100 °C.

At higher temperatures, dielectric loss spectra are dominated by long-range charge transport, i.e., dc conductivity. However, at this temperature range, the microphase separated nature of the materials gives rise to Maxwell–Wagner–Sillars interfacial relaxations [47,55,56,57,58,59], i.e., relaxations of the charges trapped at the interfaces between regions of different conductivity values. Indeed, the Maxwell–Wagner–Sillars relaxation is clearly visible as a step at the low-frequency side of the spectra (Figure 9a), superimposed on an upturn at low frequencies due to electrode polarization effects. With increasing segment molar mass, the step migrates to lower frequencies.

In order to facilitate the study of the Maxwell–Wagner–Sillars relaxation (MWS), we followed a procedure put forward by Wübenhorst and VanTurnhout [60]. A “conductivity-free” approximation of the imaginary part of the dielectric function is calculated on the basis of Kramers–Kroning relationships as follows:

(2) $$\varepsilon_{der}^{\prime \prime }=-\frac{\pi }{2}\frac{\partial \varepsilon \prime }{\partial \ln f}$$

effectively transforming the steps of ε′(f) to peaks, which are more easily detected with the naked eye and the fitting algorithms.

It is visible now that the main MWS relaxation is accompanied by a faster, much weaker, relaxation. In a previous work, we named this relaxation “g” and provisionally attributed it to a second kind of interface, possibly involving crystalline hard microdomains (Figure 9b). With increasing segment molar mass, this relaxation becomes progressively broader and more difficult to discern with the naked eye.

**Figure 10:**
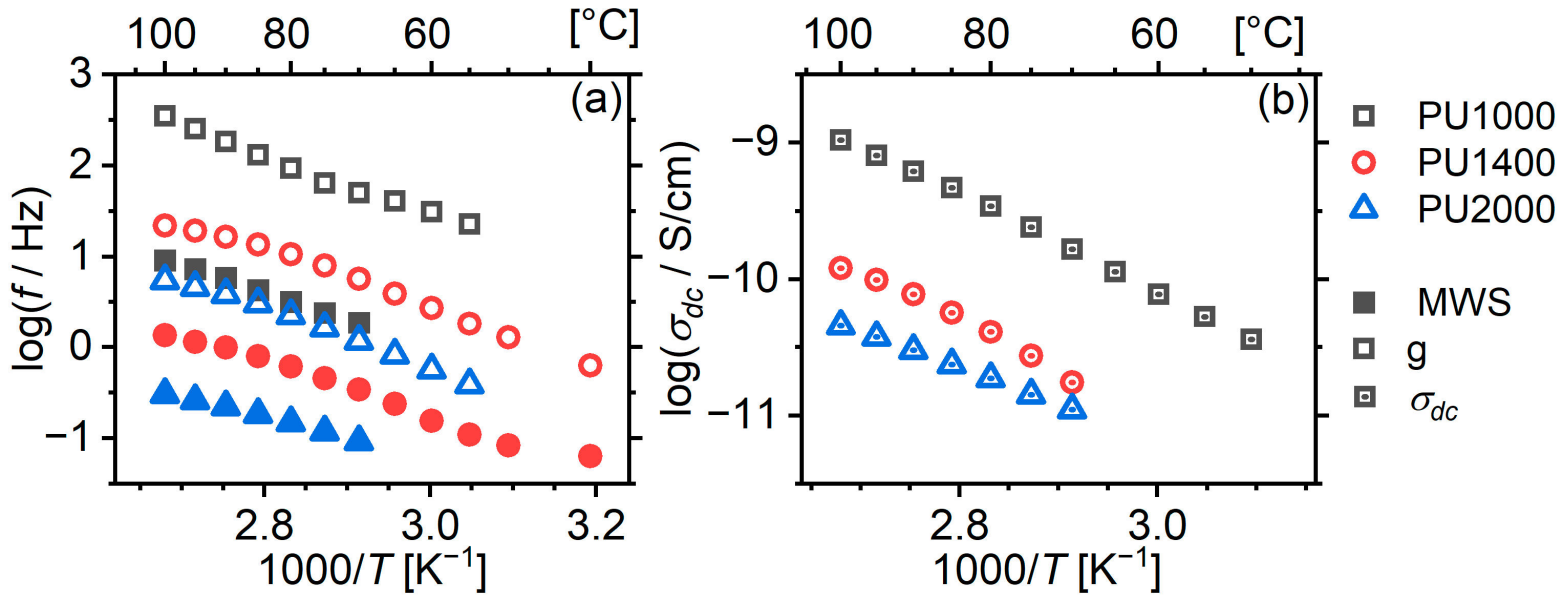
Arrhenius plot for charge-mobility-related phenomena. (**a**) interfacial relaxations; (**b**) dc conductivity.

In order to study the relaxations in more detail, we fitted Cole–Cole model functions on them (Equation (1) appropriately modified to correspond to the derivative of ε′(ln⁡f)). Both relaxations show a concave trace (Figure 10a) indicating that both processes are collaborative. In particular, matrix PU1000 shows a change in slope around 80 °C, indicating a possible change in morphology associated with the endothermic event observed via DSC several degrees below.

The long-range charge mobility was studied in terms of the dc conductivity $\sigma_{dc}$, which was quantified by fitting a term of the type:

(3) $$\varepsilon \prime \prime (f)=\frac{\sigma_{dc}}{\varepsilon_{0}\cdot 2\pi f}\;$$

on the dielectric loss spectra at high temperatures, along with a Cole–Cole peak to account for the MWS relaxation. The resulting values show a rather linear behaviour on the Arrhenius plot (Figure 10).

Our observations regarding the behaviour of the dynamics of interfacial relaxations and dc conductivity are in full agreement with what was observed in our previous work [21].

With increasing segment molar mass, $\sigma_{dc}$ decreases by approximately an order of magnitude (Figure 10b). This should be associated with the degree of phase separation and the resulting changes in the composition and amount of the soft phase. The dissolution of more hard segments in the soft phase increases its volume fraction and decreases the obstacles imposed on the mobility of charge carriers. Meanwhile, both interfacial relaxations migrate to lower frequencies with increasing segment molar mass, reflecting this change in dc conductivity.

### 2.3. Effects of POSS on Glass Transition

#### 2.3.1. Calorimetric Glass Transition

**Figure 11:**
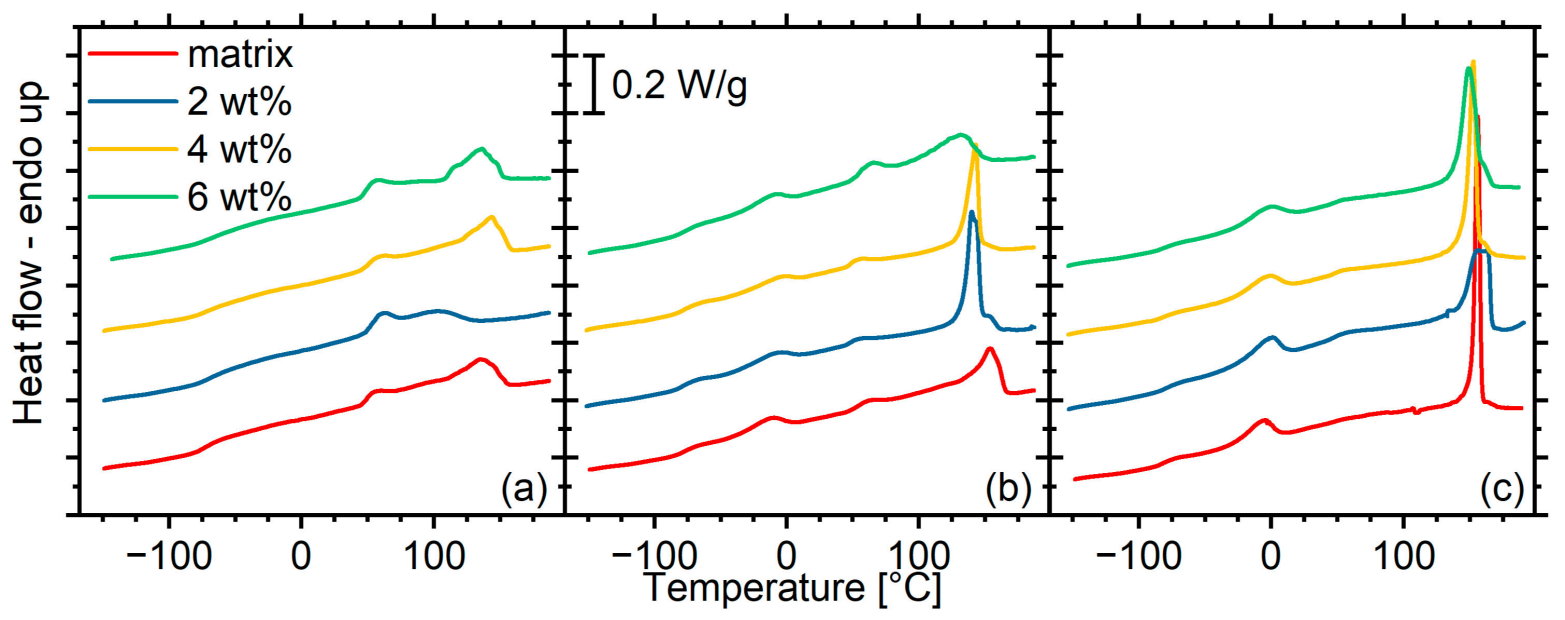
DSC curves recorded with all materials under investigation. The curves are grouped by matrix: (**a**) PU1000, (**b**) PU1400, (**c**) PU2000.

The DSC curves recorded with the composites reveal that POSS have a strong effect on the micromorphology of the materials, albeit this effect does not show clear trends (Figure 11). For the material with the shortest segments, 2 wt% of POSS results in a migration of the order/disorder transition peak above 100 °C to lower temperatures, while its intensity decreases. This indicates a morphology with a lower degree of microphase separation and with smaller hard microdomains. Higher loadings result in a similar morphology to that of the matrix. For the matrix with segments of molar mass 1400, the peak initially becomes sharper and stronger with increasing POSS content, indicating a homogenization of the size of hard microdomains, although at 6 wt% loading, the peak becomes broader and weaker, and migrates to lower temperatures. For materials with longer segments (PU2000), the peak remains sharp at all loadings, although at 2% loading, it shows a second component, and with increasing POSS content, it migrates to lower temperatures, indicating again a reduction in the size of the hard microdomains. 

**Figure 12:**
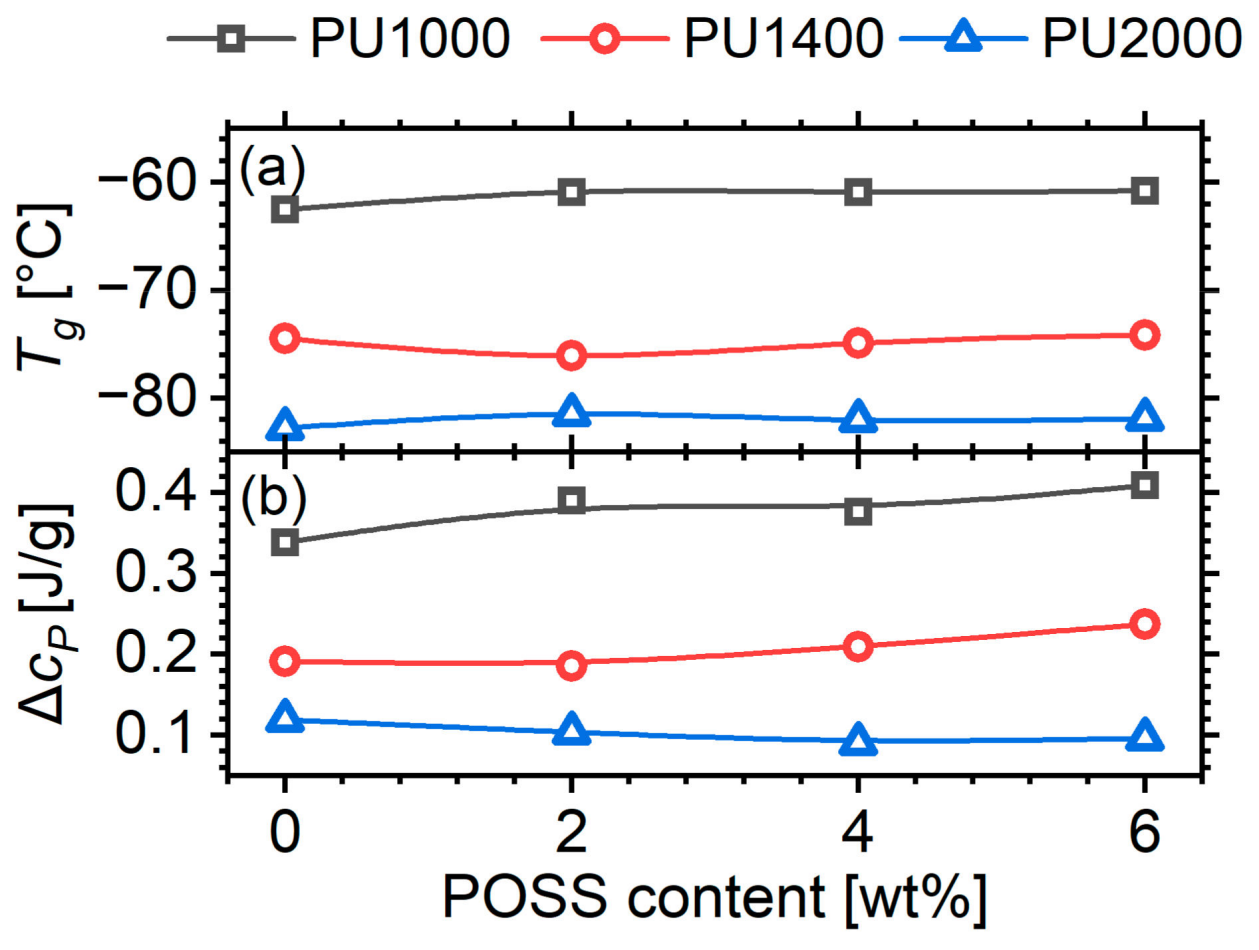
(**a**) Glass transition temperature $T_{g}$ and (**b**) heat capacity change at $\Delta c_{P}$, as a function of POSS content for the three segment molar masses.

Despite these quite significant changes in the micromorphology, the calorimetric glass transition is practically unaffected by the POSS content, both in terms of the glass transition temperature $T_{g}$ and the corresponding heat capacity change (Figure 12). This is a rather unexpected result. An increase in $T_{g}$ would be expected due to the significant crosslinking in the soft phase, as was observed in the system prepared with a rigid aromatic hard segment [24]. In this latter case, 6 wt% of POSS in a matrix with segments of a nominal molar mass of 1400 caused an increase in the glass transition temperature by approximately 10 K, which was attributed partially to crosslinking and partially to a radical suppression of the phase separation. Here, the degree of phase separation does not seem to decrease in a systematic manner, and the dissolved hard domains are not as rigid as in the previous system. A study of the dynamic glass transition via dielectric spectroscopy in the next paragraph may shed some more light on the matter.

#### 2.3.2. Dynamic Glass Transition

**Figure 13:**
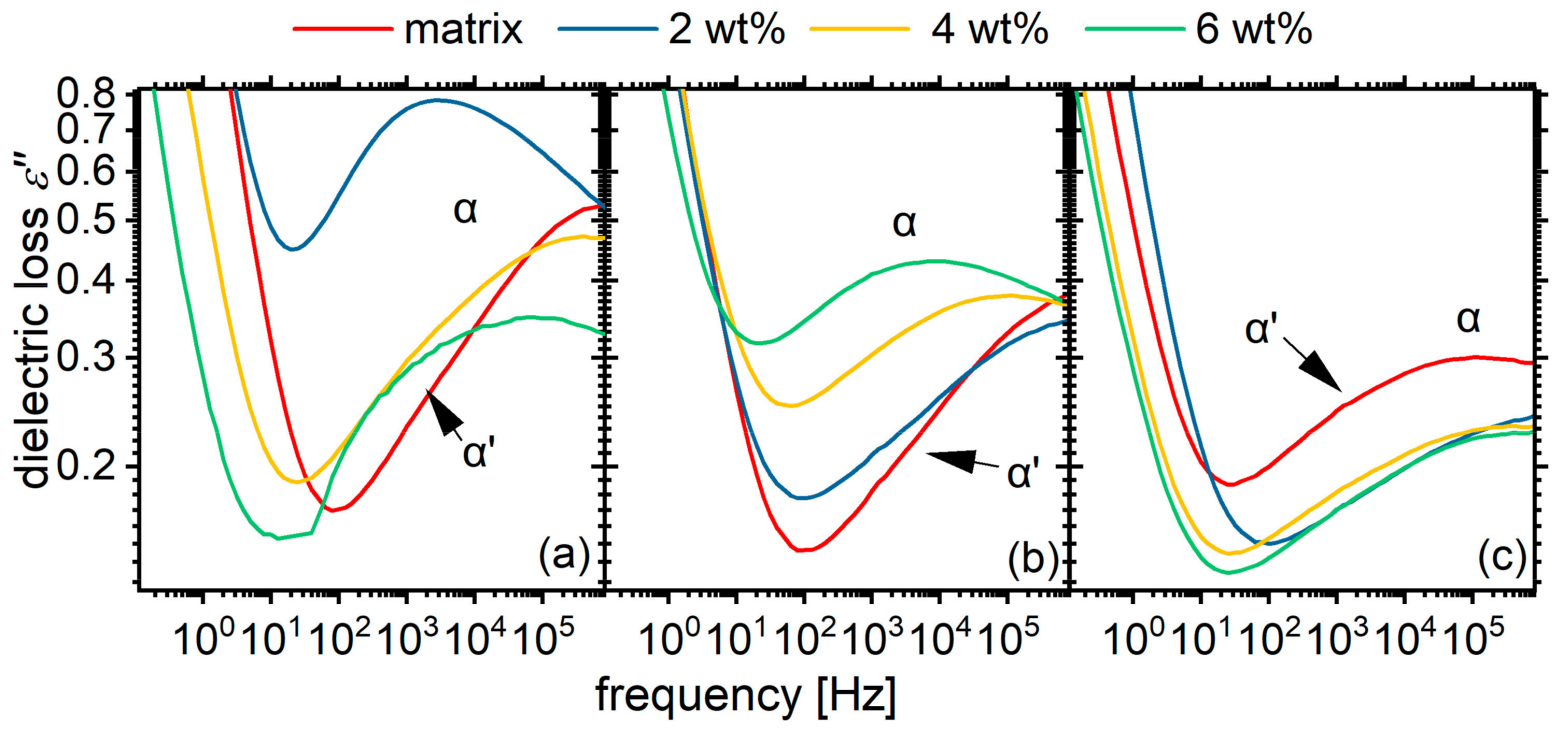
Comparative dielectric loss spectra for all materials under investigation at temperature of 5 °C, at which the low frequency side of α relaxation peak is best visible. The spectra are grouped by matrix: (**a**) PU1000, (**b**) PU1400, (**c**) PU2000. Approximate locations of the α and α’ relaxations are annotated on the plot.

The lack of effect of POSS on the calorimetric glass transition is not reflected on the dynamic one as studied via dielectric spectroscopy. The dielectric loss spectra show significant differences with varying POSS contents (Figure 13). In the material with the shortest segments (PU1000), the overall response in the region of segmental dynamics (α and α’ relaxations) migrates to significantly lower frequencies with increasing POSS content, while the low-frequency shoulder seems to be enhanced. The 2 wt% hybrid does not fit this trend. Instead, it forms a significantly asymmetric peak with no indication of a separate low-frequency shoulder. We remind our reader here that this is also a material with a significantly different micromorphology as revealed via DSC. A slowing trend is more clearly observed for the matrix with intermediate segments (PU1400). Here, at POSS contents of 4 and 6 wt%, we have an asymmetrical shape without indications of an α’ shoulder. For the materials based on the PU2000 matrix, the mobility seems rather to migrate to slightly higher frequencies with increasing POSS content.

In order to quantify these effects, we performed a standard fitting procedure as with the matrices. However, the approach with two Cole–Cole terms (Equation (1)) was not able to describe the spectra of some of the materials, e.g., the 2 wt% hybrid of matrix PU1000. In these cases, the single visible peak was strongly asymmetric; thus, a single Havriliak–Negami term was fitted to the data:

(4) $$\varepsilon^{*}=\varepsilon^{\prime }-i\varepsilon^{\prime \prime }=\varepsilon_{\infty }+\frac{\Delta \varepsilon }{{\left(1+{\left(i\frac{f}{f_{0}}\right)}^{a}\right)}^{b}}$$

This equation is essentially the Cole–Cole (Equation (1)) model modified by the exponent b in the denominator. The exponent b accounts for the asymmetry, and is equal to 1 for symmetric relaxations (essentially reducing the model to Cole–Cole). b takes smaller values for asymmetric relaxations that are broader on the high-frequency side. The peak frequency now does not coincide with $f_{0}$ but is given by the following [61]:

(5) $$f_{max}=f_{0}{\left(\frac{\sin\left(\frac{\left(1-a\right)\pi }{2+2b}\right)}{\sin\left(\frac{\left(1-a\right)b\pi }{2+2b}\right)}\right)}^{\frac{1}{1-a}}$$

**Figure 14:**
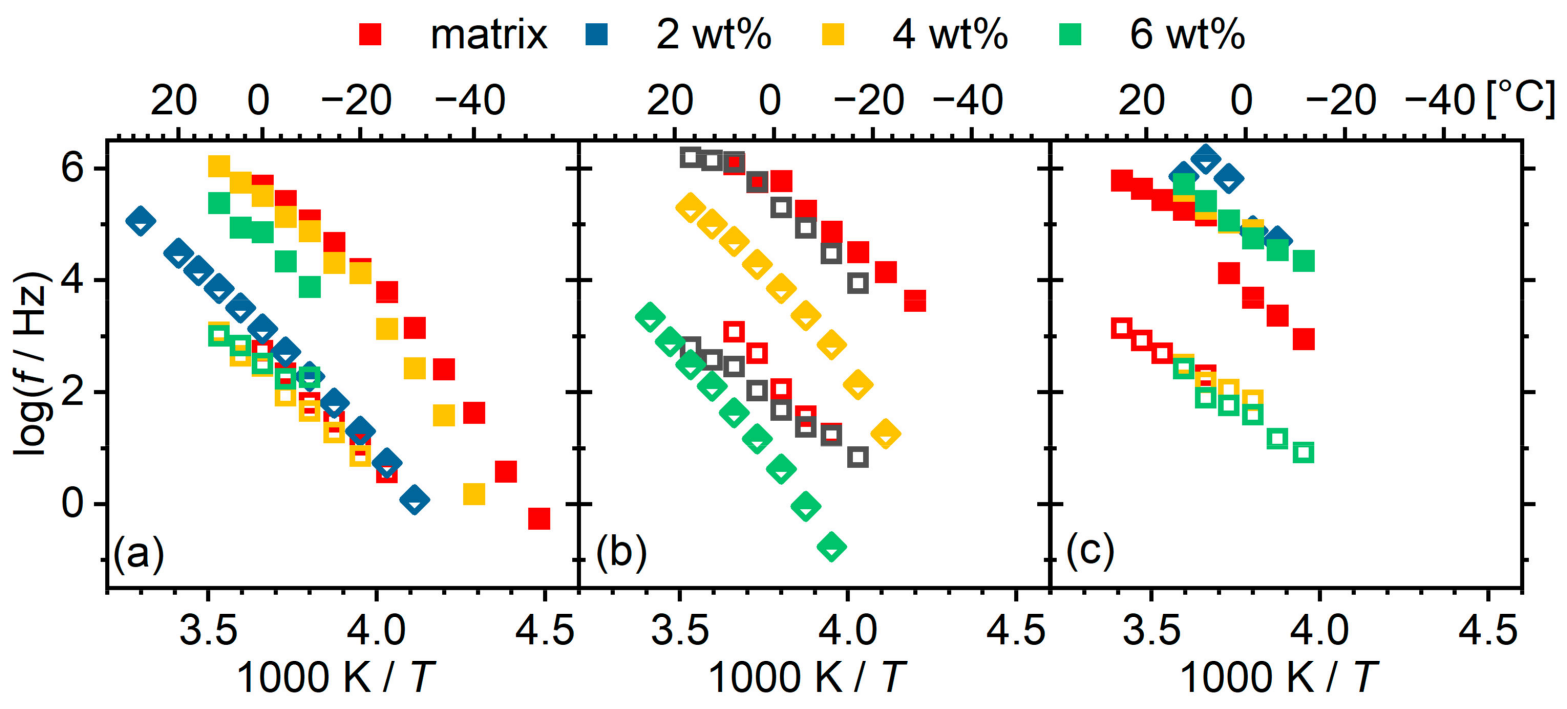
Arrhenius plots for cooperative dynamics for all materials under investigation grouped by matrix: (**a**) PU1000, (**b**) PU1400, (**c**) PU2000. POSS content indicated by colour as per the legend. Squares indicate fitting with 2 Cole–Cole functions and diamonds indicate fitting with a single Havriliak–Negami function, according to the legend. Note that the spectra of 2 wt% hybrid of matrix PU2000 was fitted with a single Cole–Cole model function due to intense overlap with neighbouring phenomena. Details are provided in text.

A representative fitting is shown in Appendix A (Figure A1). The obtained values of $f_{max}$ are plotted in the Arrhenius plot of Figure 14. The results do not show clear trends. Starting from the low-segment-molar-mass materials, the α relaxation migrates to lower frequencies with increasing POSS content (as would actually be expected), whereas the α’ remains unchanged. For the material with 2 wt% POSS, the single Havriliak–Negami relaxation is located very close to the trace of the α’ relaxation of the other materials. As we pointed out on the basis of the DSC curves, this hybrid material has a different morphology than that of the others, presumably with smaller hard domains and thus a higher number of interfaces. These interfaces are not necessarily “sharp” ones, as we have hypothesized in earlier works, but the dynamics may vary smoothly between the interface and the bulk soft phase. In the intermediate material, the change from two distinct to one asymmetric relaxation happens in a monotonic way, and the overall dynamics indeed slows down considerably and monotonically with the increasing POSS content. At the highest segmental molar mass (2000), the POSS even seems to have an accelerating effect on the α relaxation.

These observations and in particular, the discrepancy between the stability of calorimetric $T_{g}$ and the quite significant changes in the dynamic one are not easy to interpret. The following factors in synergy could affect the final outcome:

(i) Like many other properties, the glass transition in polyurethanes is strongly affected by the microphase separation. In turn, the microphase separation is affected by the compatibility of the components of the system and the conditions during its development. Here, the microphase separation was developed isothermally during the post curing phase. The morphology is very sensitive even to minor changes in the temperature or viscosity of the system during this phase. Here, the POSS, acting as a crosslinker, did not seem to have a well-defined effect on the morphology and thus on the molecular mobility. The resulting differences in the microphase separation have not only quantitative but also qualitative effects on the distribution of relaxation times in the materials.
(ii) At least in the PU1400 and PU2000 matrices, the calorimetric glass transition is studied in a temperature range at which the soft phase is partially crystalline, whereas the dynamic one is studied only after it is melted. This constitutes a significant change in the micromorphology.
(iii) We have hypothesized that in PUs based on aliphatic hard segments’ “slower” modes, whether they constitute a standalone relaxation or the dominant part of a distribution of relaxation times, have a weak thermal response. Hence, the response of the glass transition as seen via DSC depends solely on the “faster” bulk modes, which are in general not affected by the morphology of hard microdomains.

**Figure 15:**
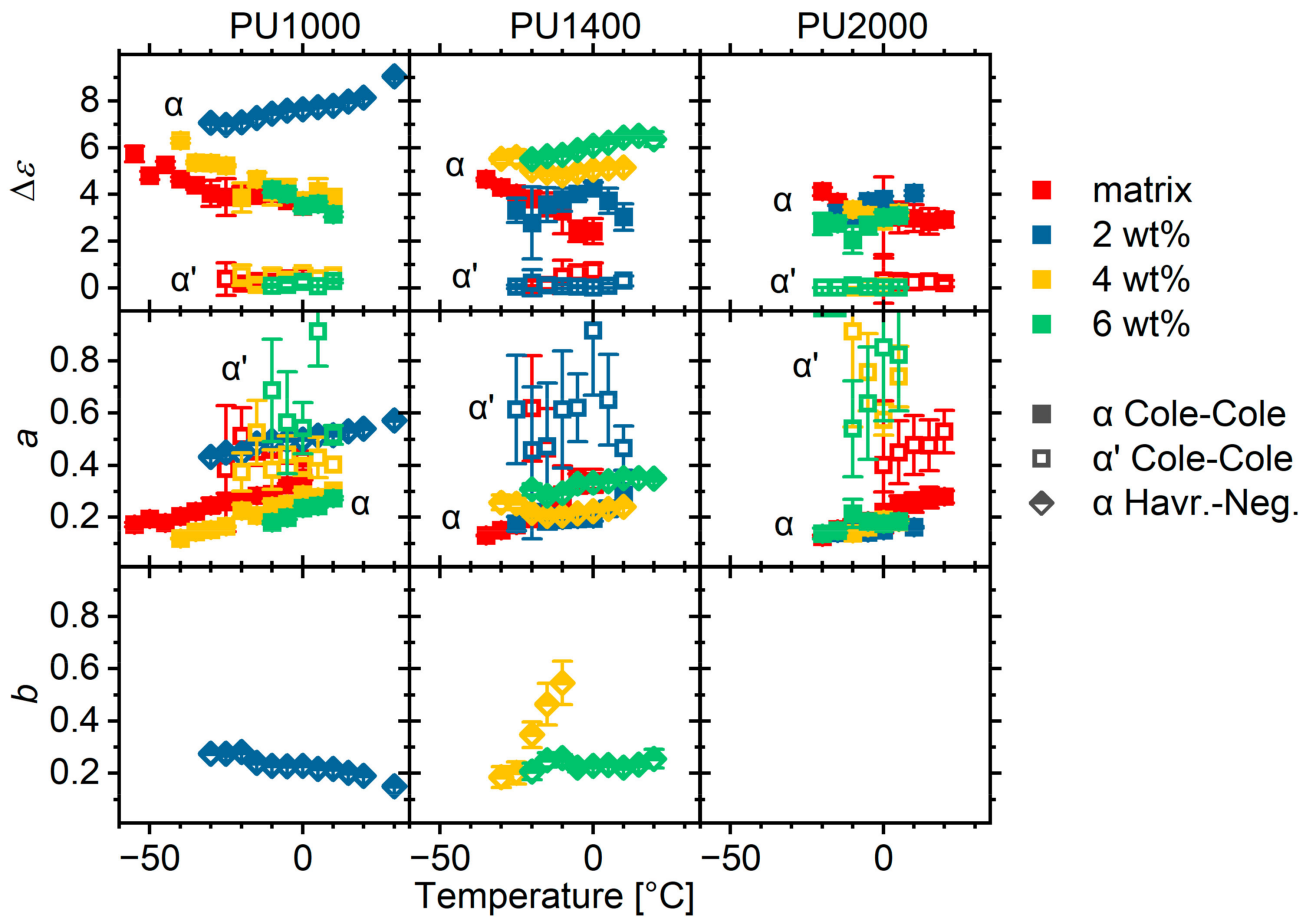
Strength Δε, broadening exponent a, and asymmetry exponent b (where applicable), for all materials under investigation. The materials are grouped by matrix according to labels on the upper part of the figure. Note that the spectra of 2 wt% hybrid of matrix PU2000 was fitted with a single Cole–Cole model function due to intense overlap with neighbouring phenomena. Details are provided in text.

The magnitude Δε of the segmental relaxations is plotted as a function of the temperature in the first row of Figure 15. With the exception of materials exhibiting asymmetric α relaxations, the POSS does not seem to have any effect on the values of the α or α’ relaxations. Materials with asymmetric relaxations seem to have larger Δε values than those of the other materials and exhibit an increasing trend with temperature. We associated the asymmetry with smooth transitions between immobilized hard segments and ones diluted in the soft phase. The higher polarizability could possibly be associated with the better polarizability of this transition layer as compared to that of immobilized hard segments.

The broadening exponent a is interpreted in terms of the heterogeneity in mobility and smaller values are associated with a broader distribution of relaxation times. The exponents of the α’ relaxation show significant scattering (Figure 15, second row); therefore, we will not comment on them. Increasing the POSS content seems to increase the heterogeneity in matrix PU1000, which should be attributed to the crosslinking. Materials with asymmetric relaxations tend to have larger a exponents; however, this should be interpreted only as a more homogeneous mobility in the long-relaxation-times side (slow modes), as the distribution of relaxation times is quite wide on the high-frequency side as indicated by the very small asymmetry exponent b (Figure 15, third row).

## 3. Discussion

So far we have shown that the glass transition in polyurethane matrices with aliphatic, “flexible” hard segments follows the established paradigm of standard polyurethanes prepared on the basis of bulkier and more rigid aromatic diisocyanates [16]. Mainly, the degree of microphase separation is established to play a key role in the glass transition. 

However, there are a few key differences. PUs based on flexible diisocyanates exhibit a higher degree of microphase separation than their aromatic counterparts. As a result, the calorimetric glass transition is significantly lower, approaching the one of the bulk polyether used as the soft phase. Despite that, the distribution of relaxation times in the dynamic glass transition is much broader, indicating a higher level of heterogeneity. This, in turn, points to a more substantial participation of diluted hard segments in the collective dynamics. In addition, in the PUs with flexible hard segments and their hybrids with POSS, a discrepancy is very commonly observed between the calorimetric and the dynamic glass transition in this and in earlier works [28]: the trends observed for the calorimetric glass transition temperature are very often not observed by the time scale of the dynamic glass transition. For example, in the case at hand, the material with longer segments has a slower dynamic glass transition than its shorter-segment counterparts, but a significantly lower glass transition temperature. Changes in the soft segment crystallinity between the temperature ranges the two phenomena occur in and radical changes in the fragility (cooperativity) of the relaxation cannot account for this observation. A plausible explanation, which we have resorted to in the past [28], is that slower modes, presumably related to segments with a higher concentration of hard segments, have a stronger dielectric but a weaker thermal response as compared to their more mobile, polyether-rich segments. Here, however, the discrepancy may be related with the complex morphology of the systems, as described in the next paragraph.

The hard domains are semi-crystalline as opposed to the fully amorphous aromatic ones. Interestingly, for a segmental molar mass of 2000, the degree of crystallinity is high enough to provide a sharp melting peak instead of several broad microphase-mixing ones, as is the case for shorter segments. This difference in the internal morphology of hard microdomains is very likely reflected in the interaction with the soft phase and thus the molecular mobility of the latter. Moreover, the soft phase also shows significant crystallinity, with a melting temperature between the glass transition temperature and the temperature region in which α relaxation is observable; i.e., the calorimetric and dynamic glass transitions are studied in different morphological states of the system.

In the system at hand, an endothermic event around 50 °C resembles a glass transition step, presumably associated with the glass transition of the hard microdomains, in agreement with the value reported for a homopolymer synthesized with HDI and butanediol [18]. In the case of systems based on rigid hard domains, at the same location, an endothermic peak appears, coined the “annealing peak” [4,17]. One of the hypotheses for its origin is that it corresponds to the enthalpy relaxation associated with the glass transition, the step of which is not visible due to having a negligible $\Delta c_{P}$ [19,20]. The presence of a step here, where the hard segments have enough mobility, corroborates this hypothesis. Interestingly, this step vanishes for high segmental molar masses, despite the more complete phase separation, which is on par with the assumption that the hard domains are almost fully crystalline at high molar masses.

In the current work, the α relaxation associated with the dynamic glass transition is accompanied by a slightly slower α’ one. In the past, evidence has been provided that it is related to confined dynamics at the interfaces between the soft phase and structures with negligible internal mobility, such as hard microdomains, nanoparticles, or polyether crystallites [16,21,23,30]. In agreement with this interpretation, in the case at hand, the relaxation is not affected by the molar mass of the segments.

Interestingly, α’ relaxation was not detected in a very similar system in the past [28]. In this earlier work, the incorporation of POSS moieties in the system resulted in a significant increase in the asymmetry of the α relaxation. This was interpreted as a continuous transition of dynamics, starting off as slow and close to the “flexible” hard domains, where the concentration of hard segments is higher, and gradually becoming faster in the bulk polymer. Again, a discrepancy was interpreted in the context of differences in the thermal and dielectric response. In the work at hand, the incorporation of POSS resulted in the transformation of the bimodal (α accompanied by α’ relaxation) form of segmental relaxation to a single asymmetric one. Interestingly, there was no monotonic behaviour with the POSS content, but the transformation was correlated with the different morphology of hard domains as reflected on the order/disorder transition endotherms at high temperatures. This ambiguity regarding the presence of α’ or the asymmetry of α points to its strong correlation with morphology which is an interesting question for future work.

The incorporation of POSS as chemical crosslinks in the soft phase has practically no effect on the calorimetric glass transition temperature, despite some non-monotonic effects on micromorphology. This is in full disagreement with the radical effects observed in a system based on an aromatic diisocyanate [24]. In the aromatic system, POSS caused a marked increase in $T_{g}$, as a result of a suppression of microphase separation. In the aliphatic system in this work, the degree of microphase separation does change. Despite this, and adding to the observations in other systems with flexible hard segments regarding the discrepancy between calorimetric and dynamic glass transitions, the incorporation of POSS leads to a non-monotonic variation in the time scale of the dynamic glass transition as observed via dielectric spectroscopy. This is associated with different microphase separations induced by POSS in a non-monotonic manner. It seems that in these particular systems, the calorimetric glass transition is sensitive to the bulk soft phase, whereas the dielectric dynamic glass transition is sensitive to the soft phase at interfaces. This point is worth following in future work. And another interesting point to follow up on is the effect of POSS on the development of microphase separation. 

## 4. Materials and Methods

The synthesis of the materials followed the prepolymer approach used in our previous work [28]. Here, the only difference is the type of POSS introduced in the matrix. Briefly, the procedure is as follows: predetermined amounts of POSS and PTMG were mixed together at temperature of 100 °C and the isocyanate was added mixed with Sn(Oct)_2_ as catalyst. Upon the end of prepolymerization, determined by stability of attenuated total reflexion–Fourier transform infrared (ATR-FTIR) spectra, 1,4, butanediol was added as chain extender in molar amount of 1.05 times the unreacted isocyanate groups, as determined by a standard back-titration. Post curing run was conducted at 110 °C for 16 h. ATR-FTIR spectra of the final materials showed that the isocyanate band at 2250 cm^−1^ vanished in the final material; hence, no unreacted isocyanates exist in the system.

Differential scanning calorimetry (DSC) experiments were performed with a TA (New Castle, Delaware, US) Q200 differential scanning calorimeter, purged with helium, calibrated by indium, and cooled by a liquid nitrogen system. A single run was performed by cooling samples of 5–10 mg enclosed in standard aluminium pans to −120 °C and heating them up to 200.

Dielectric relaxation experiments were performed with a Novocontrol (Montabaur, Germany) Alpha analyser coupled with a Quatro cryosystem of the same manufacturer. Samples with diameter of 20 mm and thickness of 1–2 mm were used. Spectra were recorded in the range of −150–100 °C, at intervals of 5 or 10 K, and in frequency range of 100 mHz to 1 MHz.

## Data Availability

Data are available on request from the corresponding author.
